# User-Centered Design of a Tablet Waiting Room Tool for Complex Patients to Prioritize Discussion Topics for Primary Care Visits

**DOI:** 10.2196/mhealth.6187

**Published:** 2016-09-14

**Authors:** Courtney R Lyles, Andrea Altschuler, Neetu Chawla, Christine Kowalski, Deanna McQuillan, Elizabeth Bayliss, Michele Heisler, Richard W Grant

**Affiliations:** ^1^ Center for Vulnerable Populations Division of General Internal Medicine University of California, San Francisco San Francisco, CA United States; ^2^ Kaiser Permanente Division of Research Oakland, CA United States; ^3^ University of Michigan Department of Veterans Affairs Ann Arbor, MI United States; ^4^ Institute for Health Research Kaiser Permanente Colorado Denver, CO United States; ^5^ Department of Internal Medicine and Health Behavior and Health Education Unversity of Michigan Ann Arbor, MI United States

**Keywords:** primary health care, chronic disease, computers, handheld, mobile applications, medical informatics, health communication

## Abstract

**Background:**

Complex patients with multiple chronic conditions often face significant challenges communicating and coordinating with their primary care physicians. These challenges are exacerbated by the limited time allotted to primary care visits.

**Objective:**

Our aim was to employ a user-centered design process to create a tablet tool for use by patients for visit discussion prioritization.

**Methods:**

We employed user-centered design methods to create a tablet-based waiting room tool that enables complex patients to identify and set discussion topic priorities for their primary care visit. In an iterative design process, we completed one-on-one interviews with 40 patients and their 17 primary care providers, followed by three design sessions with a 12-patient group. We audiorecorded and transcribed all discussions and categorized major themes. In addition, we met with 15 key health communication, education, and technology leaders within our health system to further review the design and plan for broader implementation of the tool. In this paper, we present the significant changes made to the tablet tool at each phase of this design work.

**Results:**

Patient feedback emphasized the need to make the tablet tool accessible for patients who lacked technical proficiency and to reduce the quantity and complexity of text presentation. Both patients and their providers identified specific content choices based on their personal experiences (eg, the ability to raise private or sensitive concerns) and recommended targeting new patients. Stakeholder groups provided essential input on the need to augment text with video and to create different versions of the videos to match sex and race/ethnicity of the actors with patients.

**Conclusions:**

User-centered design in collaboration with patients, providers, and key health stakeholders led to marked evolution in the initial content, layout, and target audience for a tablet waiting room tool intended to assist complex patients with setting visit discussion priorities.

## Introduction

Complex patients with two or more concurrent health conditions represent almost a third of Americans, including 80% of those aged 65 or older, and this number is growing rapidly as we face an increasingly aging and chronically ill population [1]. Complex patients make up almost three-quarters (71%) of total health care spending in the United States, and patients with multiple conditions face a variety of poorer outcomes such as decreased quality of life and increased mortality [2-5].

A large body of evidence suggests that improving patient-provider communication during primary care visits can lead to better clinical outcomes for complex patients with chronic illness. For example, patients with diabetes who see providers with shared decision-making styles are more likely to receive appropriate screening tests [6], and those who report high provider communication ratings have better self-care behaviors [7], physical and mental functioning, and glycemic control [8]. However, the current health care setting, with short face-to-face visits and high numbers of competing demands that require discussion, presents significant challenges for both patients and providers to achieving high-quality communication during visits [9,10]. Particularly for complex patients who make multiple shared decisions with their provider during the same visit, limited time during visits presents a significant obstacle to effective communication [11].

Given these challenges, our team sought to design a technology solution to facilitate visit communication between complex patients and their primary care providers. Mobile health tools like tablets offer a well-matched strategy for improving communication in a way that minimizes impact to the clinician workflow and better utilizes patients’ time in clinic [12]. Tablets in the waiting room are being increasingly used to (1) educate patients about specific health topics and (2) collect patient-reported outcome data to inform care [13]. We focused on designing a tablet app to assist patients with prioritizing their top health concerns for their next in-person visit to optimize the limited time patients and providers have together. To achieve this goal, we employed a user-centered design process to create a tablet tool for visit discussion prioritization that would be used in the clinic waiting room. We also assessed key stakeholder opinions to plan for broader dissemination of the tablet tool once built because we were well aware of the challenges in widespread adoption of technology within existing clinical settings [14]. We outline here the main findings from this qualitative work with a focus on the specific design changes that emerged from our user-centered design approach.

## Methods

Funded by a 3-year contract from the Patient-Centered Outcomes Research Institute, we planned a clinical trial intervention to help complex patients more effectively prepare for time-limited primary care visits using a tablet-based waiting room tool. The creation of this tool in the first year of the project was informed by user-centered design methodology. Borrowing from the fields of industrial and human factors engineering, user-centered design involves understanding the needs, values, and abilities of users to improve the quality of users’ interactions with and perceptions of the technology [15,16]. In practice, this involves creating a technology-delivered solution based on iterative direct user input to improve the ultimate usability of the final product before it is implemented in a real-world setting.

**Figure 1:**
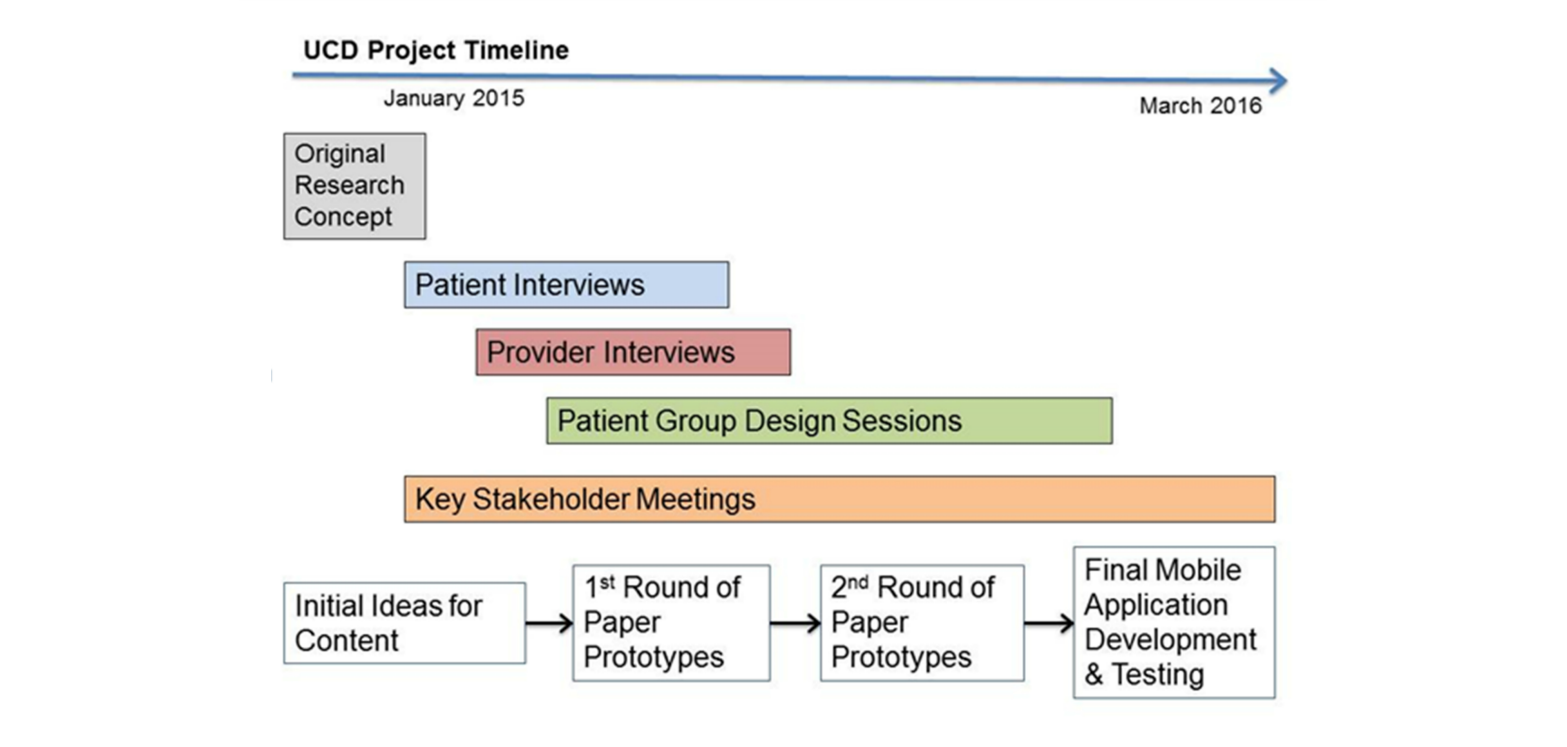
Stages of the user-centered design process.

Our user-centered design process included close work with three distinct stakeholder groups: complex patients; their primary care providers; and key leaders in health communication, education, and health technology implementation within a large integrated delivery system. We used three types of qualitative data collection approaches: one-on-one interviews with patients and providers, patient focus groups, and key informant discussions (discussed in detail below). Figure 1 outlines the steps that take the original funder research concept to the final product—through several stages of the user-centered design process.

### Study Setting

The patient and providers who participated in interviews represented primary care practices in three large health care systems across the United States: Kaiser Permanente Northern California, Kaiser Permanente Colorado, and University of Michigan, Ann Arbor. We partnered across institutions to ensure diverse representation that was not limited to a single geographical area. All of these health care systems care for large populations of medically complex patients, and investigators at each site invited patients and providers from multiple primary care practices to participate in the study. The second phase of the iterative design process (ie, focus groups and key informant meetings) was held exclusively within Kaiser Permanente Northern California, which was the primary research and implementation site for this project.

### In-Depth Interviews With Patients and Providers

In early 2015, we conducted in-person interviews with 40 patients and their 17 primary care providers: 12 patients and 5 providers from Kaiser Permanente Northern California, 13 patients and 8 providers from Kaiser Permanente Colorado, and 15 patients and 4 providers from the University of Michigan, Ann Arbor. All patients were diagnosed with two or more existing chronic conditions. Patients were selected as being medically complex by their primary care providers to ensure a high level of medical need among the sample, but the specific diagnoses of all patients were not recorded.

Patient interviews each lasted approximately 45 minutes and covered the following topics: (1) current planning processes for primary care visits, (2) usual experiences in setting a visit agenda with primary care providers, (3) interest in using a tablet in the waiting room, (4) how helpful it would be in setting their priorities for the visit, (5) what kind of support they would need before using the tablet for the first time, and (6) as the tablet was developed, what if anything about the content they would change. Providers of these patients were also interviewed regarding areas of commonality and differences in how visits should ideally be conducted and to provide specific feedback on the design of a tool to meet their workflow needs.

### Patient Focus Groups

In the second phase of the project, we conducted three 60-minute focus group sessions. These sessions were held at Kaiser Permanente Northern California using the same recruitment criteria outlined above (N=12, with repeat attendance by most patients). The primary goal of these focus group sessions was to revise a paper-based prototype of the tablet tool in which patients were shown printed screenshots of the tablet content to elicit their direct input as the design unfolded.

### Key Informant Discussions

We met with key informants throughout 2015 to similarly iterate the design of the tool, and we took detailed notes at each meeting to document the suggestions made for the tool. These meetings at Kaiser Permanente Northern California included Medicine Chiefs Steering Committee, Division of Research Information Technology team, the Permanente Medical Group technology group, Technology Multi-Media design team, the Regional Health Education team, Technology User Testing group, Communication Consultant group, and the Senior Advisory Council. We used these key informant sessions to identify design issues related to implementation and dissemination of the tablet tool, alignment with health care system priorities, and internal design and use case standards.

The stakeholders were also critical in the prototype testing of the tablet app in its various iterations, giving feedback on first the paper-based prototypes and then helping us to conduct the final prototype testing of the app once the programming was complete. More specifically, we provided key staff in the Technology User Testing and Technology Multi-Media teams with tablets with versions of the tool loaded. These individuals (in addition to 3 key research team members) clicked through all screens and available selection choices in a thorough user testing and evaluation of the more final tablet product.

### Analysis

All interview and focus group discussions were audiorecorded and professionally transcribed for analysis. For the multisite interview analyses, 3 coders (one at each site: NC, DM, and CK) collectively identified all comments relating to the design of the tablet tool and conferred with a fourth coder (CRL) to isolate patterns across sites. For the focus group analysis, all design and content modification recommendations that emerged from the group design sessions were reviewed and then categorized collaboratively by 2 members of the research team (CRL and RWG).

We organized the overall themes separately from the patient interviews, provider interviews, and patient focus groups, generating both major design theme categories and subtopics from all three sets of data. We also identified a series of exemplary quotes that reflected the topic and subtopics.

### Implications for Tablet Design

We documented major changes we made to the tablet’s design and content, informed by the user-centered design themes uncovered in our qualitative work. During this process, we used the meeting notes from the key informant discussion process in combination with the qualitative data to map the evolving tool content and design over time. As a part of this process, we saved the iterations of the prototypes to document changes as the design emerged.

## Results

### Interviews and Focus Groups

The patients in the baseline interviews across the three sites represented a mix of age, sex, and races/ethnicities. At Kaiser Permanente Northern California, the mean age was 66 (range 41-86), 67% (8/12) were female, and 67% (8/12) were white. At Kaiser Permanente Colorado, the mean age was 73 (range 57-84), 77% (10/13) were female, and 92% (12/13) were white. At the University of Michigan, the mean age was 70 (range 42-92), 40% (6/15) were female, and 87% (13/15) were white. Principal patient one-on-one interview themes are shown in Table 1. Patients were concerned about the level of technology proficiency needed to use the tool and gave specific advice about making the tool easy to use, such as adding audio instructions to walk patients through the content. They also had many specific recommendations for types of discussion topics that the tablet could trigger or prompt patients to select as they prepared for the visit, such as listing chief complaints/symptoms as well as a place to write down a sensitive issue that might be difficult to bring up. Finally, they suggested groups of complex patients for whom the tool would be most useful, stating that those who were already well organized or had productive communication with their primary care provider would not likely benefit from the tool. Despite broader comments where patients highlighted different workflows across the various clinic sites, there were not major differences in the tablet-focused themes reported here.

In parallel with the patient interviews, we asked the primary care providers of interviewed patients to answer similar questions about the agenda and priority-setting process (Table 2). Providers gave additional feedback about the content categories for a tablet tool, echoing the patients’ sentiments that this tool may be able to elicit more embarrassing or sensitive issues. These issues could otherwise be overlooked because the tool itself was viewed as a way to normalize sensitive topics as common for many patients. In addition, the providers specifically recommended the tablet as a medium to deliver brief education to patients about bringing up the top concerns to be addressed at the beginning of the visit to ensure the best use of the encounter time. Providers also suggested specific types of patients for whom the tool would be most relevant and mentioned new patients or those who recently switched their primary care providers as an important audience. Finally, providers preferred that we limit to two the number of priorities that could be highlighted by patients. To accommodate this limit, we made clear in the first brief coaching video (of two) that these two topics were simply to help start the visit and that patient could always bring up additional concerns as needed.

**Table 1:** Patient interview findings.

Category	Subtopic	Example quote(s)
Challenges for patients with limited technology proficiency	Patient lacks skills to use a tablet	“I finally bought an iPad not too long ago and I still don’t even know how to use that. I don’t know much about computers.” “Yeah, I’m not sure how many elderly people can use an iPad. I will ask you that, because I have friends who are my age or older. They don’t have a computer. They’re not computer-savvy. They don’t want to learn.”
	Suggestions to make tablet easier to use	“Can [the tablet tool] be like something like [what I have] on my phone: you have ‘speak’ and ‘talk’?” “Well, if you eliminated the writing part [for entering information into the tool], it’s okay.”
Patient recommendations for content of Pre-Visit Tool	Elicit simple lists of ongoing or new problems	“What am I here for?” You know, “What is my major complaint?” “I don’t mind checking when you give me a pad and it says ‘Check off what’s wrong with you.’”
	Provide a way to bring up topics patients might overlook	“Maybe [I want to see on the tablet tool] some things that I wouldn’t think of that would be related to what I’m dealing with.” “I may need, like, you know, a trigger: Oh, yeah, you know, I did want to ask about this.”
	Provide opportunities for personalized information/education	“It depends on how the information is presented to a patient… then it could be an educational experience.” “Yeah, after you teach them how to use it… If I knew what I was doing, you know. If somebody tells me.”
	Provide a safe place to bring up sensitive topics	Patient would add to the list of topics on the tablet: “Are there any issues you're dealing with that maybe you’re hesitant to bring up?” “You know there’s some things that you don’t, even with, even with Dr. [PCP] you know that you may not feel comfortable bringing out and maybe if you could write it down that maybe actually helpful in your treatment.”
Recommendations for which patients should not receive tool	Those who are very stable	“I could see how people who don’t really know what is wrong could find that useful, but my conditions have been the same for 15 years now. Nothing has really changed.”
	Those with clear communication with provider already	“I’m doing all that right now by the email. And if he [my doctor] reads them, he knows what I’m talking or thinking about.”

**Table 2:** Provider interview findings.

Category	Subtopic	Example quote
Provider recommendations for content of Pre-Visit Tool	Prioritize the most important health concern(s)	“Let’s get your most important issue out first.”
	Helps with sensitive issue discussions	“If they got to write it [a sensitive topic] down, they maybe potentially feel more comfortable letting it flow.”
	Want to see the entire list of patient concerns	“I like to actually see it [the whole list] so I know, you know, how much is there that we’re going to have to get through?”
Recommended role in preparing patient for time-limited encounter	Help set expectations/priorities	“I want you to choose which is priority to you today, because I don’t think I can go through all these problems today.”
	Help patient focus on long-term health issues	“Too much time spent on ‘urgent & not important,’ not enough time spent on ‘not urgent & important.’”
	Emphasize the importance of next steps/follow-up	“Think about what your health goals might be going forward…a lot of times the patients have not thought about it.”
Recommendations for which patients should receive tool	Some patients need more time to complete it	“It would probably work better if [my patients] had it at home somehow and they could complete it and then either bring it with them or send it in advance.”
	Especially helpful for new patients	“But, I am having brand-new patients added every day and, really, honestly, sometimes, this focus [on their top priorities for the visit] is very important.”

Focus group participants were primarily women (n=9, 75%) and an average age of 71 years. For the focus group sessions, patients reviewed iterative versions of the tablet tool prototype. At this stage in the design, the groups gave very specific feedback about the length of time to complete, the simplicity of wording used, and the final layout (Table 3). As one example of wording changes, we originally used the language of “documenting the care plan” as a goal for patients and providers to work on during the visit. However, patients thought this meant the type of insurance coverage they had rather than the treatment decisions made during the visit, and we therefore renamed this to “plan for your treatment,” which was clearer.

**Table 3:** Patient focus group feedback.

Category	Example quote
Shorten tool length	“I like to just zip through things, and just give me the basics. Don’t extrapolate.”
	“That’s what I’m talking about. I guess getting to the short point of it.”
Simplify/clarify wording	“I’m focusing in on what I’m hoping to get from this visit, and I wouldn’t know what ‘form filled out’ meant.”
	Most preferred terminology of “concerns” over “issues.”
	They didn’t like describing the tool as “private” (as it doesn’t mean much in this day and age), but stating that the information “will not be shared with anyone” is clearer.
	Other word suggestions included the use of first person: “we” or “us.”
Improve the layout	“I need to have something visual. I’m not a hearing learner. I’m a visual person.”
	They felt that skipping should be possible in case people didn’t want to fill something out
	They gave positive feedback about having the doctor’s picture on the first page with the patient’s name: “You gave it some thought. You know my name.”
	Instead of a progress bar, they suggested “Page 2 of 5” or “Step 1 of 5.”
	They needed more clarity about the conclusion of the tool: “Patients need to know what to do going forward”; “Say something like ‘Please share this with your doctor’ or ‘Remember to bring up this list at the beginning of your visit.’”

### Key Stakeholder Meetings

During the stakeholder meetings, we identified additional design areas that could be modified. Stakeholder groups provided essential input on the need to combine text with video to reinforce patient learning and to employ first-person pronouns to help engage patients. They also suggested allowing patients to choose videos at the start of tool by offering several options that showed individuals of different sexes and races/ethnicities, which also increased the personalization of the patient experience when using the technology.

One of the other major findings from the key informant meetings was the critical importance of both executive and clinical leadership support to enable more seamless implementation of the tool into existing clinical workflows. More specifically, the buy-in achieved in these meetings allowed us to consider other provider workflow considerations for use of the tool in real-world practice and to jumpstart the Spanish language adaptation of the tablet app to expand the reach of the project for a larger patient population.

Finally, the stakeholder prototype testing allowed us to identify long lists of potential bugs and incorrect sequencing of information to fix on the more final version of the tablet tool. Their assistance also allowed us to verify that the information entered by patients was being populated and stored correctly on our servers. These final prototyping steps were critical to the launch of the subsequent randomized trial phase of this project.

### Design Changes to the Tablet Tool

**Figure 2:**
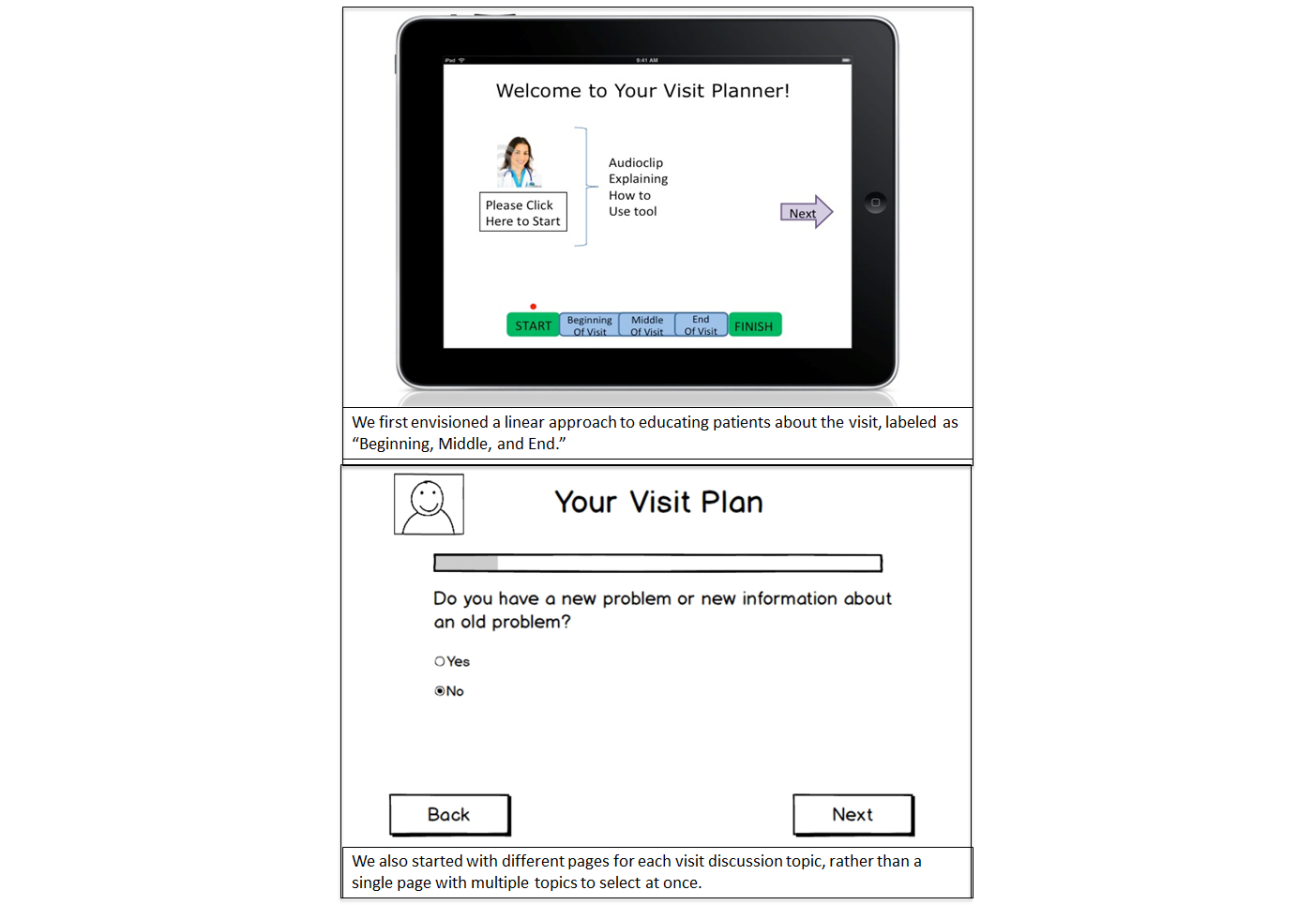
Early prototypes of the tablet tool.

**Figure 3:**
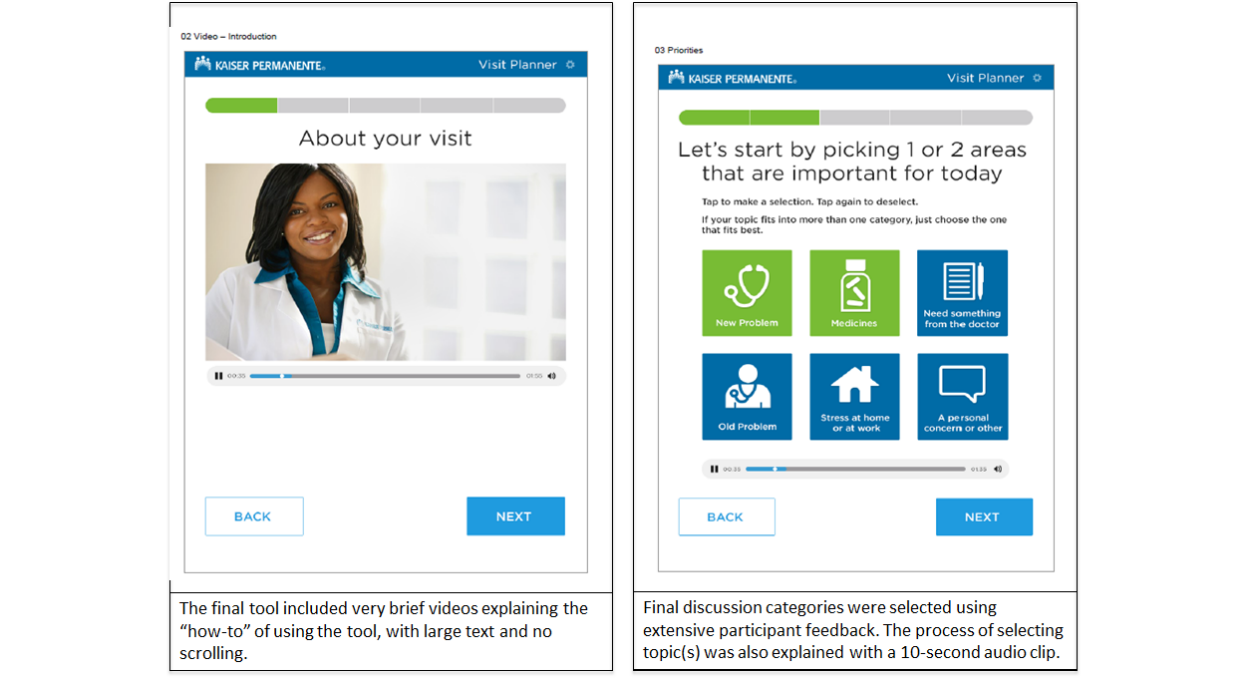
Later prototypes of the tablet tool.

These qualitative findings were used in concert with the key informant meeting results to inform major design decisions to the tablet tool, some of which are visually represented in Figures 2 and 3 displaying early versus later prototypes of the tablet tool. First, we made several changes to make the tool more accessible, especially for older adults without tablet experience. We removed all scrolling from the app, increased the size of the font and the choice buttons even more than anticipated, and worked continuously to edit text and keep it below a 7^th^ grade reading level.

Next, to capture the full educational potential of the tool, we embedded two <30-second video recordings to give patients guidance about the importance of (1) bringing up discussion priorities at the beginning of the visit with their doctor and (2) making a plan with the doctor for the next steps after the visit. Participants could pause these videos or skip them altogether based on their individual preference. Videos featured a health provider speaking directly to the patient. To appeal to a wider range of both visual and auditory learning styles, these videos also had brief text intermittently appear on the screen to underscore the key points of the video. These audiovisual elements also reduced the overall reading burden of using the tool. Finally, based on participant feedback, we made the decision to offer headphones to all participants to make the experience private while listening to the videos in the waiting room and to have wipes accessible to clean off the tablet in between users.

Based on our iterative design process with patients, physicians, and other stakeholders, our final six discussion topic choices on the tool also improved. We used the feedback from participants outlined above to ensure that “new problems/symptoms” were made distinct from “old problems/symptoms” and that an open-ended “personal concern” choice was prominent to allow space for patients to bring up sensitive topics as needed. In response to additional provider feedback, we added a discussion topic choice of “Need something from the doctor” in order to address the administrative requests from patients for referrals or other paperwork, and we limited the number of allowed choices to the patient’s one or two top priorities.

Importantly, we cut out significant portions of script text as we went through the design process. While we started out with a relatively short app, we continued to reduce the information presented at every step. The final version of the tablet tool contained three sections with a maximum of six screens that included sufficient space for free text entry. Most of the information on each screen that was deleted from the original version of the tool was explanations about “why” the tool could be helpful, leaving simply the “how-to” information as the core messages presented. For example, early prototypes envisioned using the tool content to walk patients through the beginning, middle, and end of their visit with their provider—that is, to help them prepare to raise their concerns at the beginning, engage with care planning during the middle portion of the visit, and then leave the visit with concrete action items for follow-up. However, we realized throughout our process that patients would be better served with straightforward prompts eliciting their discussion priorities, followed by tips for staying engaged during and after the visit—without extraneous details about when or why they might use this information when communicating with their provider. This time-agnostic approach was much more flexible for applying to all encounters, including visits with less predictable scenarios not following a typical linear timeframe.

Finally, we expanded the target audience for the tablet tool. We originally designed and tested the tool for complex patients: those identified by their providers as needing additional time or assistance with visit discussion prioritization. Our user-centered design findings identified another target audience: new patients, either to the health care system or switching to a new primary care provider. Moving forward to the next randomized trial phase of this project, we made a decision to focus patient recruitment on both of these groups who might need this tool.

## Discussion

### Principal Findings

Through the use of user-centered design methods, we substantially modified our initial design concept based on robust input from patients, physicians, and key informant leaders within a large integrated delivery system. The final tool was simpler to use while also providing richer choices and more engaging layouts and was better suited for widespread implementation within the health care system.

Our findings are significant because user-centered design methods are largely underdeveloped within the health care literature [17,18]. The data on the efficacy of health technologies are limited in part because health technology studies often report on the overall effectiveness of the technology once tested rather than describing the scientific process of incorporating structured user feedback during the design phase [14]. This relative lack of attention to user-centered design in the published literature may help explain why there is overall very mixed evidence of the benefit of health technologies within real-world clinical practice [14,19]. That is, technology studies that report a null effect on outcomes could attribute the failure of the technology to a lack of health behavior change rather than basic design flaws that resulted in poor fit of the technology to meet the end user’s needs. Furthermore, our findings highlight the need to conduct user-centered design with complex patients with multiple chronic conditions, as most interventions and technologies are targeted to single diseases or health behaviors [20].

Similar to previous studies, our design and testing process found that using a combination of methodologies (in our case several different types of qualitative inquiry) produced more robust results than a single user-centered approach alone [21,22]. For example, if we had relied on only patient one-on-one interviews, we would have missed key provider perspectives about how to focus in on their new patients for whom the tool could be particularly beneficial. Furthermore, the layout-specific prototype feedback during the patient focus groups and the key informant meetings identified key areas where changes in the design could remove extraneous information and thus reduce patient confusion.

We also included a diverse patient population with complex health care conditions in this user-centered design work that better reflected the general patient population with multiple chronic conditions [23]. This diversity in participants ensured a wide range of feedback about the tool, as opposed to only recruiting participants who had prior experience using tablets. This was a particularly relevant aspect of our design process that helped us make concrete decisions about simplifying the text presented in the tool and incorporating several audiovisual features to enhance patient comprehension. Use of multiple modalities (text, video, audio) and including flexibility to skip sections allowed us to create a tool that could be used by patients with varying levels of comfort with technology or with absorbing new information. These changes are more likely to make the final tool accessible for a broader target audience, which has implications for wider implementation in future work.

### Limitations

There are several limitations to note. First, we recruited patients from three large integrated delivery systems, which may not be representative of other health care settings. Second, we did not do more formal usability testing (such as with validated usability ratings) [18], relying instead on more general prototype iterations to improve the layout. While additional methods may have further enriched our findings, we decided from the outset that multiple user groups of patients, providers, and key informants were the most critical to the design process in our setting.

### Conclusions

Our study rigorously documented findings from our tablet tool design, which is a critical step of health informatics research that can produce generalized knowledge about the user-centered design process. Future mHealth research should combine several design and usability testing methods in health technology development, as well as document this design process. This can not only improve the technology and its ultimate impact but also disseminate key lessons learned that other developers and investigators can build on.

## References

[1] Pefoyo AJK, Bronskill SE, Gruneir A, Calzavara A, Thavorn K, Petrosyan Y, Maxwell CJ, Bai Y, Wodchis WP (2015). The increasing burden and complexity of multimorbidity. BMC Public Health.

[2] Koroukian SM, Warner DF, Owusu C, Given CW (2015). Multimorbidity redefined: prospective health outcomes and the cumulative effect of co-occurring conditions. Prev Chronic Dis.

[3] Park M, Katon WJ, Wolf FM (2013). Depression and risk of mortality in individuals with diabetes: a meta-analysis and systematic review. Gen Hosp Psychiatry.

[4] Divo M, Cote C, de Torres JP, Casanova C, Marin JM, Pinto-Plata V, Zulueta J, Cabrera C, Zagaceta J, Hunninghake G, Celli B (2012). Comorbidities and risk of mortality in patients with chronic obstructive pulmonary disease. Am J Respir Crit Care Med.

[5] Fortin M, Lapointe L, Hudon C, Vanasse A, Ntetu AL, Maltais D (2004). Multimorbidity and quality of life in primary care: a systematic review. Health Qual Life Outcomes.

[6] Heisler M, Tierney E, Ackermann RT, Tseng C, Narayan KMV, Crosson J, Waitzfelder B, Safford MM, Duru K, Herman WH, Kim C (2009). Physicians' participatory decision-making and quality of diabetes care processes and outcomes: results from the triad study. Chronic Illn.

[7] Heisler M, Bouknight RR, Hayward RA, Smith DM, Kerr EA (2002). The relative importance of physician communication, participatory decision making, and patient understanding in diabetes self-management. J Gen Intern Med.

[8] Aikens JE, Bingham R, Piette JD (2005). Patient-provider communication and self-care behavior among type 2 diabetes patients. Diabetes Educ.

[9] Parchman ML, Pugh JA, Romero RL, Bowers KW (2007). Competing demands or clinical inertia: the case of elevated glycosylated hemoglobin. Ann Fam Med.

[10] Kaplan SH, Greenfield S, Gandek B, Rogers WH, Ware JE (1996). Characteristics of physicians with participatory decision-making styles. Ann Intern Med.

[11] Kerr EA, Heisler M, Krein SL, Kabeto M, Langa KM, Weir D, Piette JD (2007). Beyond comorbidity counts: how do comorbidity type and severity influence diabetes patients' treatment priorities and self-management?. J Gen Intern Med.

[12] Patel V, Hale TM, Palakodeti S, Kvedar JC, Jethwani K (2015). Prescription Tablets in the Digital Age: A Cross-Sectional Study Exploring Patient and Physician Attitudes Toward the Use of Tablets for Clinic-Based Personalized Health Care Information Exchange. JMIR Res Protoc.

[13] Jensen RE, Rothrock NE, DeWitt EM, Spiegel B, Tucker CA, Crane HM, Forrest CB, Patrick DL, Fredericksen R, Shulman LM, Cella D, Crane PK (2015). The role of technical advances in the adoption and integration of patient-reported outcomes in clinical care. Med Care.

[14] Kumar S, Nilsen WJ, Abernethy A, Atienza A, Patrick K, Pavel M, Riley WT, Shar A, Spring B, Spruijt-Metz D, Hedeker D, Honavar V, Kravitz R, Lefebvre RC, Mohr DC, Murphy SA, Quinn C, Shusterman V, Swendeman D (2013). Mobile health technology evaluation: the mHealth evidence workshop. Am J Prev Med.

[15] Corry MD, Frick TW, Hansen L (1997). User-centered design and usability testing of a web site: An illustrative case study. ETR&D.

[16] Schleyer TKL, Thyvalikakath TP, Hong J (2007). What is user-centered design?. J Am Dent Assoc.

[17] Lyles C, Schillinger D, Sarkar U (2015). Connecting the Dots: Health Information Technology Expansion and Health Disparities. PLoS Med.

[18] LeRouge C, Wickramasinghe N (2013). A review of user-centered design for diabetes-related consumer health informatics technologies. J Diabetes Sci Technol.

[19] Kellermann AL, Jones SS (2013). What it will take to achieve the as-yet-unfulfilled promises of health information technology. Health Aff (Millwood).

[20] Zulman DM, Jenchura EC, Cohen DM, Lewis ET, Houston TK, Asch SM (2015). How Can eHealth Technology Address Challenges Related to Multimorbidity? Perspectives from Patients with Multiple Chronic Conditions. J Gen Intern Med.

[21] Lyles CR, Sarkar U, Osborn CY (2014). Getting a technology-based diabetes intervention ready for prime time: a review of usability testing studies. Curr Diab Rep.

[22] Henderson VA, Barr KL, An LC, Guajardo C, Newhouse W, Mase R, Heisler M (2013). Community-based participatory research and user-centered design in a diabetes medication information and decision tool. Prog Community Health Partnersh.

[23] Gerteis J, Izrael D, Deitz D, LeRoy L, Ricciardi R, Miller T, Jayasree B (2014). Multiple Chronic Conditions Chartbook.

